# Dietary green tea residue improves growth and antioxidant capacity via rumen microbiota and glutathione modulation in finishing beef cattle

**DOI:** 10.1186/s40104-026-01485-w

**Published:** 2026-09-02

**Authors:** Changxiao Shi, Jiajie Deng, Hongliang Zhang, Yingqi Li, Shuo Zhang, Huili Wang, Shengnan Min, Yawen Luo, Zhihao Zhang, Jun Hao, Yutao Wang, Binghai Cao, Yang He, Huawei Su

**Affiliations:** 1 State Key Laboratory of Animal Nutrition and Feeding, College of Animal Science and Technology, China Agricultural University, Beijing, 100193 People’s Republic of China; 2 School of Agriculture and Environment, College of Sciences, Massey University, Palmerston, North, 4410 New Zealand; 3 Bioeconomy Science Institute – AgResearch Group, Grasslands Research Centre, Palmerston, North, 4442 New Zealand; 4 Department of Grassland Science, College of Animal Science, Guizhou University, Guiyang, 550025 People’s Republic of China; 5 Guizhou Cattle Industry Group Co., Ltd., Guizhou, 550001 People’s Republic of China

**Keywords:** Antioxidant, Beef cattle, Glutathione, Green tea residue, Growth performance, Microbiome

## Abstract

**Background:**

Green tea residue (GTR) is an abundant by-product rich in polyphenols. This study aimed to investigate the effects of GTR on growth performance, rumen microbiota, and systemic metabolism in finishing beef cattle.

**Results:**

Forty-five 18‑month‑old finishing Angus steers with an average initial body weight of 474.44 ± 14.03 kg (mean ± SD) were individually housed and randomly assigned to three dietary treatments (*n* = 15 per group) using stratified randomization by initial body weight: control group (CG group, 150 g rice straw), low-dose GTR group (LG group, 75 g rice straw +75 g GTR), and high-dose GTR group (HG group, 150 g GTR). The feeding trial lasted 70 d with a 10-day adaptation period. To elucidate the regulatory mechanisms of GTR on beef cattle growth, we thoroughly assessed growth performance, nutrient digestibility, ruminal fermentation parameters, conducted bacterial 16S rRNA sequencing, analysed plasma biochemical and antioxidant markers, and executed an untargeted plasma metabolomic analysis. The LG group tended to increase average daily gain (ADG). Meanwhile, the LG group exhibited elevated plasma concentrations of β-hydroxybutyrate and glucose, increased activities of superoxide dismutase and glutathione S-transferase, an increased glutathione (GSH)/glutathione disulfide (GSSG) ratio, and reduced GSSG content. GTR treatment decreased reactive oxygen species and oxidative stress index. Furthermore, dietary GTR significantly modified the rumen microbial community structure, enriching *Xylanibacter* and *Prevotellaceae_UCG-003*, while reducing the abundance of *Rikenellaceae_RC9_gut_group*. Plasma metabolomics revealed that GTR treatment enriched GSH Metabolism and the Pentose Phosphate Pathway. Notably, gamma-glutamylcysteine was identified as a key mediator of the association between *Xylanibacter* and ADG in fattening beef cattle supplemented with GTR.

**Conclusions:**

Dietary supplementation with 75 g/steer/d GTR improved growth performance and systemic antioxidant capacity in finishing beef cattle by modulating the rumen microbiota and activating the glutathione system. GTR represents a promising functional feed additive that simultaneously valorizes tea by‑products and enhances livestock productivity.

**Graphical Abstract:**

**Figure:**
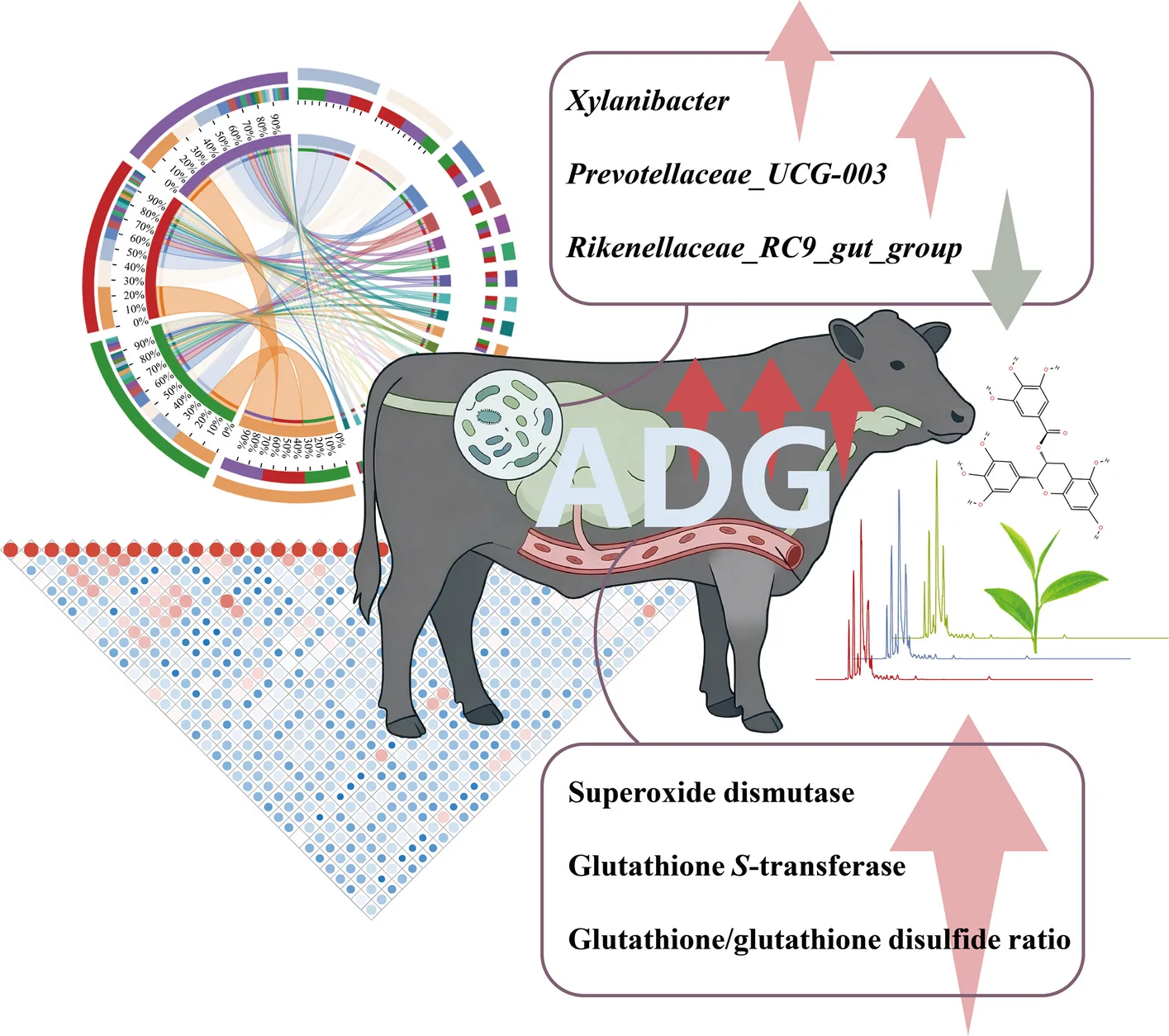

**Supplementary Information:**

The online version contains supplementary material available at https://doi.org/10.1186/s40104-026-01485-w.

## Introduction

Aligning with the One Health approach that recognizes the interconnectedness of human, animal, and environmental health, sustainable livestock farming must prioritize animal health [1]. Phytogenic feed additives have emerged as a key strategy to achieve this, offering a natural alternative to antibiotics while supporting overall animal well-being [2]. In modern beef cattle finishing systems, high-concentrate diets are widely applied to improve average daily gain (ADG) and shorten the finishing cycle. Although high-energy diets enhance production efficiency, they disrupt ruminal pH homeostasis, induce subacute ruminal acidosis, and trigger systemic oxidative stress and inflammation, ultimately impairing animal performance [3]. Plant-derived ingredients are rich in bioactive compounds such as flavonoids, which show considerable potential in enhancing the antioxidant capacity of animals [4, 5]. Among these flavonoids, tea polyphenols (TPS) are well documented to activate the endogenous glutathione (GSH) system [6]. TPS upregulate the expression of multiple antioxidant enzyme genes, such as superoxide dismutase (SOD) and glutathione peroxidase (GSH-Px), and effectively strengthens the cellular capacity to eliminate oxidative damage products [7]. Meanwhile, the tea industry generates over 5 million tons of by‑products annually in China alone, the majority of which are underutilized despite their high residual polyphenol content [8]. Repurposing green tea residue (GTR) as a functional feed ingredient for finishing cattle offers potential benefits by simultaneously reducing agricultural waste and enhancing animal health. However, the GTR’s effects and mechanisms under high-concentrate feeding conditions on ruminants remain insufficiently investigated.

Most studies on TPS or tea by-products supplementation in ruminant diets have focused on in vitro culture systems and dairy cattle, revealing their ability to modulate rumen fermentation patterns and microbial community composition, including the relative abundances of *Rikenellaceae_RC9_gut_group* and *Ruminococcaceae_NK4A214_group* [9–11]. However, previous research has reported that microbial changes have not been consistently linked to improved antioxidant status or growth performance. In monogastrics, tea by-products are well documented to upregulate the Nrf2‑GSH pathway and increase the expression of antioxidant enzymes [12, 13]. However, current findings indicate that the responses of the GSH antioxidant system to polyphenols in ruminants remain controversial [14]. This discrepancy likely reflects the dose‑dependent, dual‑edged effects of polyphenols on the rumen ecosystem. To date, it remains unclear how graded dietary supplementation of GTR modulates beef cattle rumen microbiota and subsequently the host antioxidant defense system, nor whether such microbiota-host interactions ultimately improve growth performance under high-concentrate finishing regimens.

Based on this, we hypothesized that dietary GTR supplementation improves systemic antioxidant status and growth performance through an integrated microbiota–metabolite–host signaling axis. These components may mitigate oxidative stress induced by high-concentrate feeding, with the result of regulating the animals’ antioxidant capacity and ruminal microbiota, thereby enhancing the production performance of finishing beef cattle. The present study aimed to evaluate the effects of two GTR supplementation levels on growth performance, ruminal fermentation, and plasma antioxidant indices, and to unravel the underlying microbial and metabolic mechanisms using 16S rRNA gene sequencing and untargeted plasma metabolomics. This research intends to provide a scientific basis for the high-value utilization of GTR as a sustainable feed additive in beef production.

## Materials and methods

### Animal ethics statement

This animal experiment received approval from the Animal Welfare and Ethics Committee of China Agricultural University (Approval No. AW62904202-1-1). All experimental protocols were conducted in strict compliance with the ARRIVE guidelines.

### Experimental materials

**Fig. 1:**
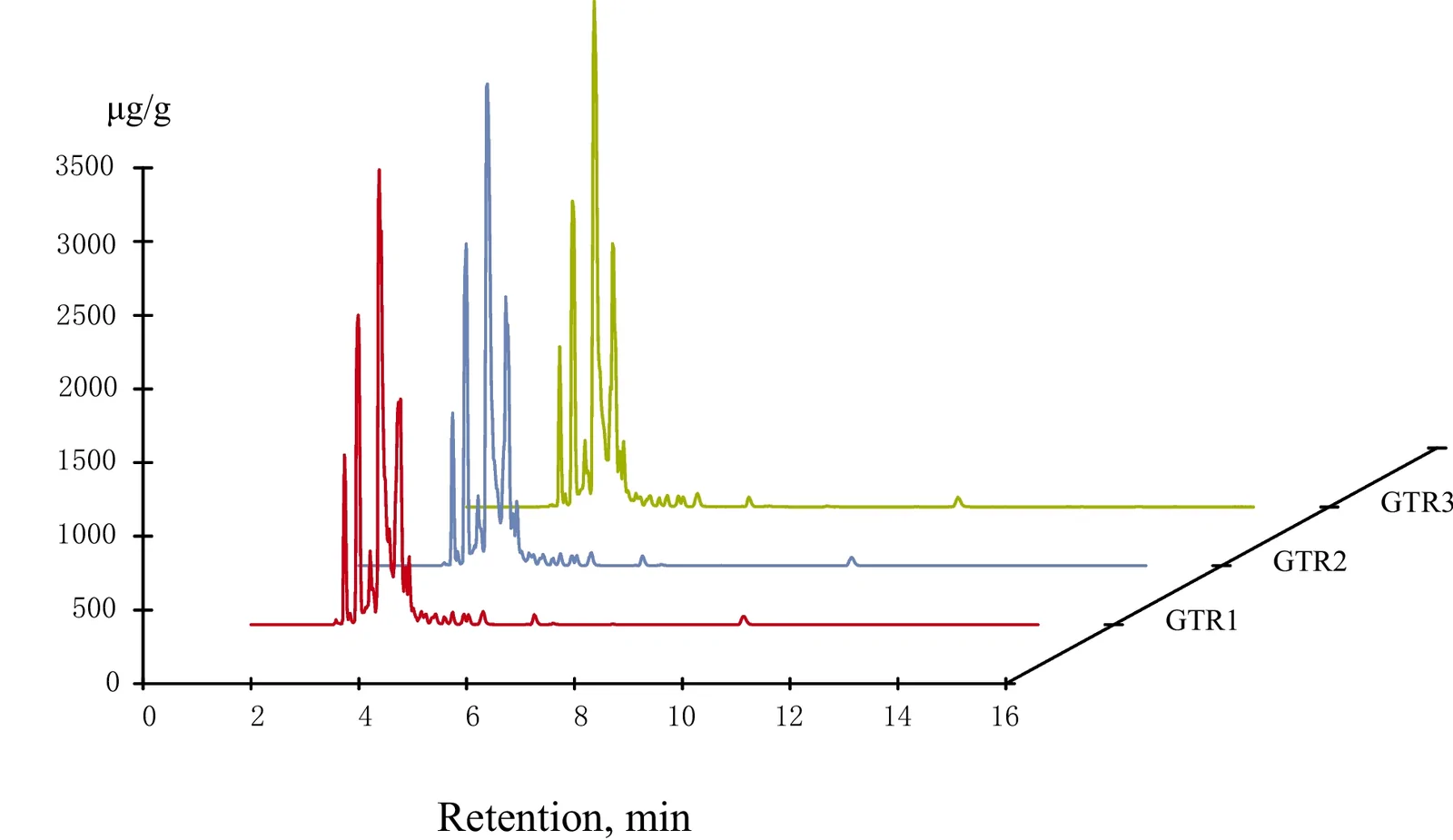
Representative total ion chromatogram of GTR with annotated peak areas and retention times (*n* = 3)

This study was conducted at the Guizhou Yanjiao Cattle Farm (Bijie, China; 105.67° E, 27.34° N), with experimental animals provided by the Guizhou Cattle Industry Group Co., Ltd. Prior to the feeding trial, we collected and analysed multiple types of tea by-products from different regions. After evaluating their TBS content, water soluble carbohydrate levels, and supply stability, we selected GTR from Guizhou Anshun Tea Industry (Group) Co., Ltd. as the experimental material. The nutritional components and flavonoid profile of selected GTR are shown in Table 1, Fig. 1 and Additional file 1.

**Table 1:** Nutrient composition and flavonoid profile (top 10) of GTR (dry matter basis)

Items	Content, %
Dry matter	93.31
Crude protein	16.29
Ether extract	3.18
Neutral detergent fiber	26.36
Acid detergent fiber	20.87
Ash	4.41
Tea polyphenols	15.59
Water soluble carbohydrates	28.23
Flavonoids composition, μg/g	
Epigallocatechin gallate	3,477.00
Gallocatechin gallate	2,623.19
(-)-Epigallocatechin	1829.54
Procyanidin B2	1502.19
(-)-Catechin gallate	340.54
Gallic acid	332.20
(-)-Gallocatechin	246.51
Theaflavin 3,3'-digallate	218.68
Theaflavin	170.02
(-)-Catechin	159.33

### Experimental design and animal management

Forty-five 18-month-old Angus steers (474.44 ± 14.03 kg, mean ± SD) were ranked by initial body weight and randomly assigned to three treatment groups (*n* = 15 per group) using a stratified randomization procedure, ensuring balanced initial body weight across treatments. The control group (CG) received 150 g of rice straw per head daily; the low-dose group (LG) received 75 of rice straw and 75 g of GTR per head daily; and the high-dose group (HG) received 150 g of GTR per head daily. Each steer was housed in a separate 5 m × 3 m pen equipped with a feeder, neck restraint, and water bowl. The pens were equipped with shade structures, and manure is removed daily. Steers were provided with ad libitum total mixed rations (TMR) daily at 0800 and 1500. All animals were free to drink water all day long. Animal diet was formulated according to NASEM, as detailed in Table 2 [15]. The trial lasted 70 d, including a 10-day adaptation period. All feeding and management practices were kept identical to those in the trial period, with only necessary sampling carried out additionally.

**Table 2:** Diet composition and nutrient composition of finishing Angus steers (dry matter basis), %

Items^1^	Contents	Chemical composition	Content
Ground corn	35.57	Crude protein	13.99
Flaked barley	5.28	Ether extract	1.33
DDGS	11.60	Neutral detergent fiber	40.06
Soybean meal	5.61	Acid detergent fiber	22.83
Corn gluten feed	4.62	Starch	28.89
Lysine by-product	2.31	Ash	8.86
Corn germ meal	1.32	Gross energy, Mcal/kg	4.15
Limestone	0.99		
Extruded urea	0.33		
1% Beef cattle finishing premix	0.66		
Sodium bicarbonate	0.99		
Salt	0.66		
Dicalcium phosphate	0.66		
Magnesium oxide	0.33		
Live yeast	0.07		
Wheat straw	29.00		

^1^The 1% beef cattle finishing premix provides a guaranteed minimum content per kilogram of the following nutrients: Vitamin A ≥ 1,050 KIU, Vitamin D₃ ≥ 360 KIU, Vitamin E ≥ 9,000 IU, Vitamin C ≥ 2,500 mg, Vitamin B-complex ≥ 700 mg, Niacinamide ≥ 2,000 mg, Biotin ≥ 28 mg; Cu ≥ 2,800 mg, Zn ≥ 9,000 mg, Fe ≥ 3,500 mg, Mn ≥ 7,500 mg, Co ≥ 55 mg, I ≥ 160 mg, Se ≥ 70 mg. The maximum moisture content is 10% on a per kilogram basis. *DDGS* Distillers dried grains with solubles

### Sample collection and analysis

#### Growth performance indexes

The TMR offered to each steer was weighed before feeding daily. The TMR was fed in batches, ensuring that the GTR was completely consumed before the remaining feed was provided to the steers. The orts were collected and weighed before the next day’s feeding to calculate actual dry matter intake (DMI). Body weight (BW) of beef cattle was weighed at the beginning and the end of the experiment before morning feeding (d 0 and 61), respectively, to determine ADG. The feed to gain ratio (F/G) was calculated as DMI divided by ADG. Feeding cost and economic efficiency were estimated based on market prices of feed and live cattle.

#### Feed and fecal sample collection and analysis

Feed ingredients and TMR samples were collected daily for three consecutive days before the end of the trial (d 58–60). GTR samples were cryogenically ground and stored at −80 °C (MM400, Retsch, Haan, Germany). At the same time points, fecal samples were collected from each animal via rectal. These samples were immediately mixed thoroughly with 10% tartaric acid at a 1:4 ratio (w/w) to stabilize nitrogen, then dried at 65 °C and ground.

In GTR, the determination of TPS was carried out following the Chinese national standard GB/T 8313–2018 [16]. The content of water-soluble carbohydrates (WSC) was measured using a colorimetric method after reaction with anthrone reagent [17]. Flavonoids were extracted from freeze‑dried, ground GTR samples (20 mg) with 0.5 mL of 70% methanol containing internal standards. After ultrasonication for 30 min and centrifugation (12,000 × *g*, 4 °C, 5 min), the supernatant was filtered through a 0.22-μm membrane and analysed on an AB Sciex QTRAP 6500⁺ LC‑MS/MS system. Chromatographic separation was performed on a Waters ACQUITY UPLC HSS T3 C18 column (100 mm × 2.1 mm, 1.8 μm) at 40 °C with a gradient of 0.05% formic acid in water (A) and 0.05% formic acid in acetonitrile (B) at a flow rate of 0.35 mL/min (0–1 min, 10%–20% B; 1–9 min, 20%–70% B; 9–12.5 min, 70%–95% B; 12.5–13.5 min, 95% B; 13.5–13.6 min, 95%–10% B; 13.6–16 min, 10% B). The mass spectrometer was operated in both positive and negative electrospray ionization modes with scheduled multiple reaction monitoring. Compound identification relied on a self‑built MetWare database (http://www.metware.cn/) constructed using authentic standards, and quantification was performed using external standard calibration curves with MultiQuant 3.0.3 software. Neutral detergent fiber (NDF), acid detergent fiber (ADF), and acid-insoluble ash (AIA) in feed and fecal samples were measured in accordance with standard protocols [18]. Ash (AOAC 942.05), dry matter (DM, AOAC 934.01), ether extract (EE, AOAC 920.39), and crude protein (CP, AOAC 984.13) were analysed according to the recommended procedures [19]. Starch content was determined using the anthrone colorimetric method [20]. Organic matter (OM) was calculated as 100 − Ash%. Gross energy (GE) was evaluated using an automatic oxygen bomb calorimeter (model 6400, Parr Instruments, Moline, IL, USA). Apparent digestibility was calculated using the AIA as an endogenous indicator [21]. The formula is as follows:

$$Apparent\,total\,tract\,digestibility\,(\%)\,=\,100\,-(Ad\times\,Nf)/\,(Af\times\,Nd)\,\times\,100$$

where Nd is the content of a given nutrient in the diet (%), Nf is the content of the same nutrient in the feces (%), Ad is the content of AIA in the diet (%), and Af is the content of AIA in the feces (%).

#### Rumen fluid sample collection and analysis

Ruminal fluid was collected via a sterilized nasogastric tube at 3 h after morning feeding on d 62 and 63 of the trial. Due to the sampling capacity, half of the steers in each group were sampled on each day. To reduce saliva contamination, the initial 200 mL of each sample was discarded. Following filtration with four layers of gauze, ruminal fluid pH was measured immediately using a Testo 205 (Lenzkirch, Germany). Subsequently, the samples were frozen in liquid nitrogen for preservation. The concentration of ammonia nitrogen (NH_3_‑N) was analysed using the phenol‑hypochlorite colorimetric method [22]. Volatile fatty acids (VFA) were quantified by gas chromatography. Detailed procedures were consistent with those reported in previous research [23]. Bacterial 16S rRNA gene sequencing was performed using the primers 338 F (5′-ACTCCTACGGGGAGGCAGCAG-3′) and 806R (5′-GGACTACHVGGTWTCTAAT-3′). Gene sequencing and subsequent bioinformatics analyses were completed with the assistance of MajorBio Biomedical Technology Co., Ltd. (Shanghai, China). Briefly, genomic DNA was extracted and the V3–V4 hypervariable region was amplified using TransStart FastPfu DNA Polymerase on an ABI GeneAmp^®^ 9700 thermal cycler, with each sample amplified in triplicate. Amplicons were purified by gel extraction, quantified using the QuantiFluor™‑ST system, pooled in equimolar concentrations, and paired‑end sequenced (2 × 150 bp) on an Illumina NextSeq 2000 platform. Raw data were quality‑filtered, denoised, and clustered into amplicon sequence variants (ASVs) using the DADA2 algorithm within the QIIME2 platform (version 2022.8). Taxonomic assignment was performed against the SILVA 138 database, followed by downstream diversity and differential abundance analyses.

#### Plasma sample collection and analysis

During weighing (d 61), blood samples were collected from the tail vein of each steer using vacuum blood tubes containing ethylenediaminetetraacetic acid. After standing at room temperature for 30 min, the samples were centrifuged at 3,000 × *g* for 15 min to obtain plasma. The plasma was then immediately frozen and stored in liquid nitrogen.

Plasma biochemical profiles, including aspartate aminotransferase (AST), total protein (TP), alkaline phosphatase (ALP), cholesterol (CHO), triglyceride (TG), high-density lipoprotein cholesterol (HDL-C), low-density lipoprotein cholesterol (LDL-C), urea nitrogen (UREA), non-esterified fatty acid (NEFA), glucose (GLU), β-hydroxybutyrate (BHB), creatinine (CREA), alanine aminotransferase (ALT), and albumin (ALB), were analysed with an automatic biochemical analyser (Hitachi 7020, Hitachi Ltd., Japan). Globulin (GLB) was calculated as: GLB = TP − ALB. All corresponding assay kits were supplied by Beijing Jiuqiang Biotechnology Co., Ltd. (Beijing, China). Plasma oxidative stress, antioxidant, and immune markers, comprising SOD, total antioxidant capacity (T-AOC), catalase (CAT), GSH-Px, GSH, glutathione S-transferase (GST), malondialdehyde (MDA), oxidized glutathione (GSSG), reactive oxygen species (ROS), interleukin-1β (IL-1β), tumor necrosis factor α (TNF-α), and interleukin-18 (IL-18), were detected via commercial kits from Jiangsu Meimian Industrial Co., Ltd. (Yancheng, Jiangsu, China). The oxidative stress index (OSI) was calculated with the formula: OSI = ROS/T − AOC. The plasma samples were subjected to untargeted metabolomic analysis using an ExionLC™ AD UPLC system coupled to an AB Sciex QTRAP 6500⁺ mass spectrometer in both positive and negative electrospray ionization modes. Metabolites were separated on an ACQUITY UPLC HSS T3 C18 column (100 mm × 2.1 mm, 1.8 μm; Waters) at 40 °C with a gradient of water (0.1% formic acid) and acetonitrile (0.1% formic acid) at 0.35 mL/min. Quality control (QC) samples were prepared by mixing equal aliquots of all test samples and were analysed periodically to monitor system stability. Raw mass data were processed for peak detection, retention time alignment, and median normalization, followed by log_10_ transformation; metabolites with relative standard deviation (RSD) > 30% in QC samples were excluded. Metabolite annotation was performed by searching against the HMDB database (http://www.hmdb.ca/), Metlin (https://metlin.scripps.edu/), and an in-house database. Subsequent bioinformatics analysis was conducted with the support of MajorBio Biomedical Technology Co., Ltd. (Shanghai, China).

### Data analysis

Raw data were organized in Excel 2019, and statistical analyses were conducted using SPSS 25.0 unless otherwise specified. A two-sided *P*-value ≤ 0.05 was considered statistically significant, and 0.05 < *P* ≤ 0.10 was considered a tendency, unless otherwise indicated for multiple-testing-adjusted analyses.

For growth performance, data were analysed using analysis of covariance (ANCOVA), with treatment as a fixed effect and the corresponding baseline measurement as a covariate. Initial body weight (d 0 BW) was included as a covariate for final body weight, ADG, and F/G. For DMI, the adaptation period average DMI was used as the covariate. The model was expressed as:

$$Y_{ij}\;=\mu+\alpha_i\;+\;{\beta X}_{ij}\;+\epsilon_{ij}$$

where *Yᵢⱼ* is the dependent variable, *μ* is the overall mean, *αᵢ* is the fixed effect of the *i*^th^ treatment, *β* is the regression coefficient for the covariate *Xᵢⱼ* (the baseline value of the respective covariate), and *εᵢⱼ* is the random residual error ~ N (0, *σ*^2^*ε*). Linear and quadratic effects of increasing GTR dose (0, 75, and 150 g/steer/d) were evaluated using orthogonal polynomial contrasts within the same model.

All remaining variables were collected once at the end of the trial. Because each animal contributed a single independent observation for these endpoints and no pre-treatment measurements were available as covariates, these data were analysed by one-way ANOVA, with treatment as the sole fixed factor. The model was:

$$Y_{ij}=\mu+\tau_i+\varepsilon_{ij}$$

where *Y*_*ij*_ is the dependent variable, *μ* is the overall mean, *τ*_*i*_ represents the effect of the *i*^th^ treatment (dietary group), and *ε*_*ᵢⱼ*_ is the residual error. When the overall F-test was significant, post-hoc pairwise comparisons were conducted using Fisher’s LSD test. Linear and quadratic effects of increasing GTR dose (0, 75, and 150 g/steer/d) were evaluated using orthogonal polynomial contrasts within the same model.

Rumen microbiota analyses were performed after ASV generation and taxonomic annotation described in Rumen fluid sample collection and analysis. Principal component analysis (PCA) was conducted with the R ropls package (v.1.6.2). Differentially abundant genera among groups were identified using the Kruskal–Wallis rank sum test, with *P*-values adjusted for multiple testing using the Benjamini–Hochberg false discovery rate (FDR) method. Spearman’s correlation analysis was subsequently conducted with the psych package (version 4.2.3) in R. Network visualization was carried out using Gephi software (version 0.10.1; https://gephi.org). Causal mediation analysis was performed with the lavaan package (version 4.2.3) in R. Metabolites were annotated by searching against the HMDB and KEGG (https://www.kegg.jp/kegg/pathway.html) databases, and pathway enrichment analysis was conducted using the scipy.stats package in Python. All figures were compiled and refined with Adobe Illustrator 2022.

## Results

### Growth performance

Feeding GTR showed a trend toward increasing the 61 d BW (*P* = 0.080) and ADG of Angus steers (*P* = 0.076), while the F/G exhibited a quadratic trend (*P* = 0.060). Feeding costs in the CG group were significantly lower than in the experimental groups (*P* = 0.005). Both ADG benefits (*P* = 0.073) and farm profit (*P* = 0.078) demonstrated a quadratic trend with the GTR inclusion dose increased. The LG group generated the highest farm profit (27.2% greater than CG; Table 3).

**Table 3:** Effects of GTR addition on growth performance and economic feasibility of finishing Angus steers

Items^1^	Group				SEM	*P*-value		
	CG	LG	HG			Treatment	Linear	Quadratic
0 d BW, kg	476.00	474.33	473.00		2.092	0.851	0.573	0.971
61 d BW, kg	544.93	552.73	543.07		2.587	0.080	0.866	0.026
DMI, kg	11.38	11.66	11.56		0.071	0.787	0.997	0.492
ADG, kg	1.17	1.31	1.14		0.031	0.076	0.654	0.027
F/G	9.88	9.15	10.45		0.254	0.100	0.297	0.060
Feeding costs, CNY/d	30.67^b^	31.94^a^	32.17^a^		0.208	0.005	0.002	0.205
ADG benefits, CNY/d	44.59	49.66	43.45		1.176	0.073	0.686	0.025
Farm profit, CNY/d	13.92	17.71	11.28		2.690	0.078	0.315	0.039

^1^*CNY* Chinese Yuan, *BW* Body weight, *ADG* Average daily gain, *DMI *Dry matter intake, *F/G* Feed to gain ratio

^a,b^Different lowercase letters indicate significant differences (*P* < 0.05)

### Apparent nutrient digestibility

As the GTR feeding dose increased, the apparent digestibility of organic matter (DOM) exhibited a quadratic trend (*P* = 0.090; Table 4).

**Table 4:** Effects of GTR addition on apparent nutrient digestibility of finishing Angus steers, %

Items^1^	Group			SEM	*P*-value		
	CG	LG	HG		Treatment	Linear	Quadratic
NDF	55.28	54.48	55.61	0.728	0.818	0.856	0.546
ADF	49.64	49.72	50.57	0.757	0.866	0.630	0.818
CP	64.38	64.19	64.58	0.786	0.981	0.921	0.867
EE	57.64	59.52	62.39	1.337	0.360	0.158	0.864
OM	65.36	68.36	66.79	0.627	0.156	0.353	0.090

^1^*ADF* Acid detegent fiber, *CP* Crude protein, *EE* Ether extract, *NDF* Neutral detegent fiber, *OM* Organic matter

### Rumen fermentation

With increasing dietary levels of GTR in Angus steers, rumen fluid pH exhibited a trend of initial decrease in the LG group followed by an increase in the HG group (*P* = 0.078). The A/P ratio tended to be altered (*P* = 0.067; Table 5).

**Table 5:** Effects of GTR addition on rumen fermentation indexes of finishing Angus steers

Items^1^	Group				SEM	*P*-value		
	CG	LG	HG			Treatment	Linear	Quadratic
pH	6.69	6.58	6.66		0.026	0.180	0.587	0.078
Acetate, mmol/L	55.90	59.49	59.60		1.449	0.523	0.320	0.587
Propionate, mmol/L	11.81	12.79	12.44		0.314	0.463	0.430	0.340
Isobutyrate, mmol/L	0.60	0.60	0.57		0.014	0.653	0.471	0.569
Butyrate, mmol/L	7.77	8.29	8.22		0.250	0.675	0.481	0.597
Isovalerate, mmol/L	1.10	1.13	1.06		0.029	0.614	0.508	0.467
Valerate, mmol/L	0.33	0.36	0.34		0.009	0.294	0.775	0.127
TVFA, mmol/L	77.51	82.66	82.22		2.009	0.538	0.360	0.530
A/P	4.75	4.65	4.79		0.030	0.158	0.584	0.067
NH_3_-N, mg/dL	18.21	18.45	16.49		1.341	0.826	0.621	0.715

^1^*A/P* Acetate to propionate ratio, *TVFA* Total volatile fatty acids, *NH*_*3*_*-N* Ammonia nitrogen

### 16S rRNA of rumen fluid

**Fig. 2:**
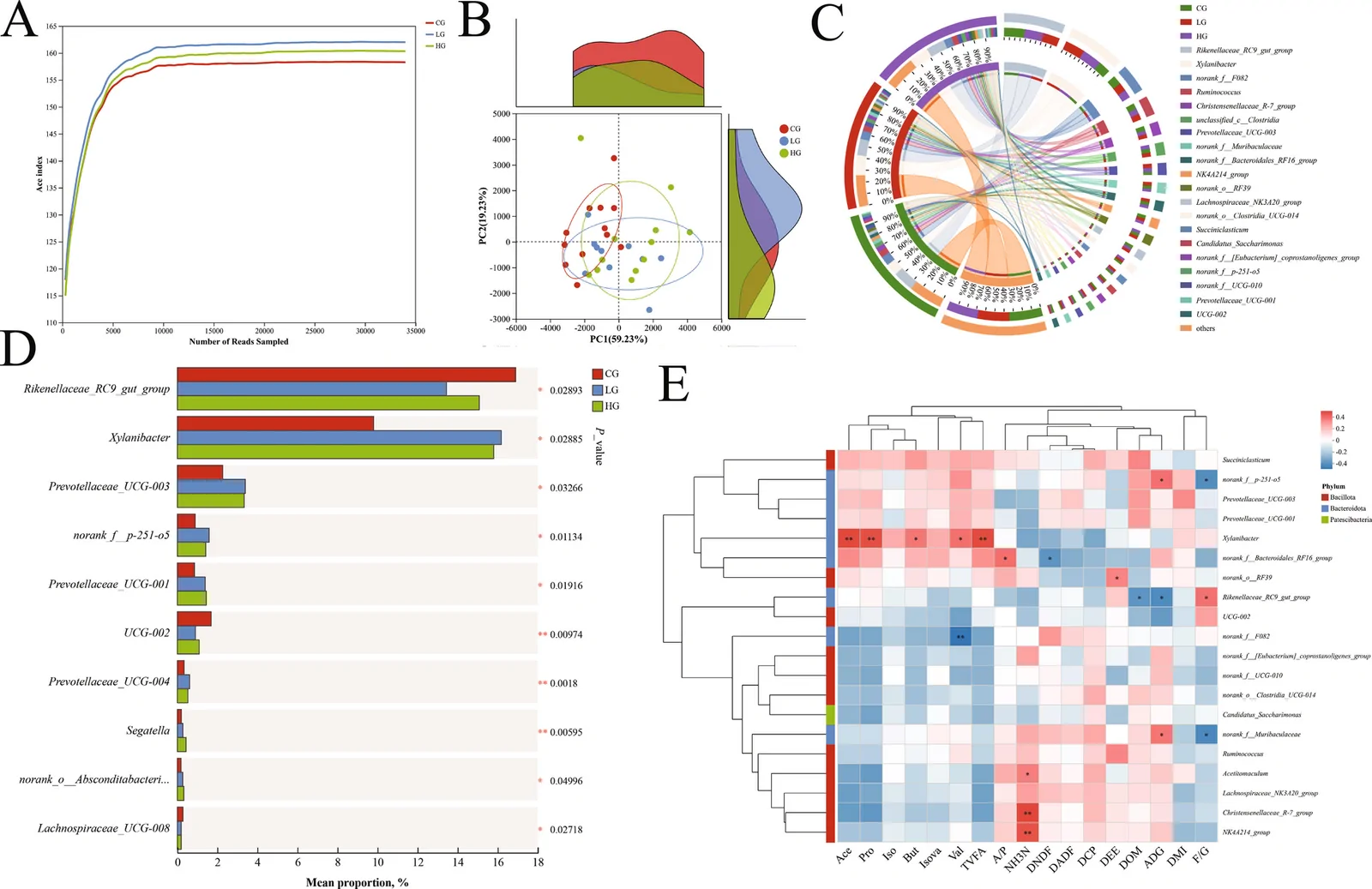
Effects of different levels of GTR on 16S rRNA in rumen fluid of finishing Angus steers (*n* = 12 per group). **A** Bacterial rarefaction curves. **B** PCA of the bacterial community. **C** Circos diagram illustrating the relative abundance of the Top 20 genera. **D** Differences in bacterial genus abundance between groups. **E** Correlation heatmap between differential bacterial genus and metabolic parameters. Significance levels: ^*^P < 0.05, ^**^P < 0.01, ^***^P < 0.001

16S rRNA sequencing generated an average of 2,971,148 raw reads per sample. The rarefaction curves plateaued with increasing sequencing depth, suggesting that the sequencing coverage adequately represented the true microbial community structure (Fig. 2A). PCA revealed a distinct separation among groups, indicating that dietary GTR supplementation significantly influenced the rumen microbial community structure in Angus steers (Fig. 2B). At the genus level, the predominant bacteria included *Rikenellaceae_RC9_gut_group*, *Xylanibacter*, *norank_f__F082*, etc. (Fig. 2C). Dietary GTR supplementation significantly reduced the relative abundances of *Rikenellaceae_RC9_gut_group* and *UCG-002*, while markedly increasing those of *Xylanibacter*, *Prevotellaceae_UCG-003*, *norank_f__p-251-o5*, and other genera (*P* < 0.05; Fig. 2D). Correlation heatmap analysis further revealed that *Xylanibacter* was significantly positively correlated with acetate, propionate, butyrate, valerate, and TVFA (*P* < 0.05). *Rikenellaceae_RC9_gut_group* showed significant negative correlations with DOM and ADG, and a significant positive correlation with F/G (*P* < 0.05; Fig. 2E). Both *norank_f__p-251-o5* and *norank_f__Muribaculaceae* were significantly positively correlated with ADG and negatively correlated with F/G (*P* < 0.05).

### Plasma biochemical parameters

The CG treatment significantly decreased plasma BHB levels relative to the groups receiving GTR supplementation (*P* = 0.021). With increasing dietary GTR inclusion, GLU, CHO, and HDL-C tended to increase (0.05 < *P* ≤ 0.10), while LDL-C showed a significant linear increase (*P* = 0.045). Additionally, TG and ALP displayed a tendency towards a linear increase (0.05 < *P* ≤ 0.10). AST displayed a quadratic trend, rising first and declining subsequently with elevated GTR levels (*P* = 0.091; Table 6).

**Table 6:** Effects of GTR addition on plasma biochemical indexes of finishing Angus steers

Items^1^	Group			SEM	*P*-value		
	CG	LG	HG		Treatment	Linear	Quadratic
BHB, mmol/L	0.20^b^	0.25^a^	0.24^a^	0.008	0.021	0.016	0.140
TG, mmol/L	0.28	0.30	0.33	0.011	0.212	0.081	0.971
GLU, mmol/L	3.07	3.35	3.08	0.054	0.055	0.979	0.017
ALT, U/L	38.56	36.14	37.38	1.658	0.849	0.782	0.620
ALP, U/L	5.18	6.58	6.97	0.400	0.167	0.074	0.552
AST, U/L	74.76	83.79	77.65	2.065	0.202	0.569	0.091
ALB, g/L	31.98	32.71	33.23	0.389	0.448	0.210	0.902
TP, g/L	58.16	61.23	59.32	0.919	0.408	0.616	0.217
GLB, g/L	26.18	28.53	26.09	0.683	0.273	0.961	0.110
ALB/GLB	1.24	1.17	1.28	0.025	0.226	0.551	0.107
LDL-C, mmol/L	0.57	0.62	0.71	0.028	0.126	0.045	0.755
CREA, mmol/L	85.15	88.08	88.71	1.692	0.677	0.412	0.757
UREA, mmol/L	5.48	5.68	5.59	0.098	0.735	0.677	0.510
CHO, mmol/L	2.82	3.28	3.52	0.126	0.070	0.024	0.667
NEFA, mmol/L	0.19	0.23	0.19	0.011	0.437	0.931	0.203
HDL-C, mmol/L	1.27	1.44	1.40	0.033	0.094	0.106	0.136
HDL/LDL	2.32	2.43	2.08	0.076	0.157	0.203	0.145

^1^*ALB* Albumin, *ALT* Alanine aminotransferase, *ALP* Alkaline phosphatase, *AST* Aspartate aminotransferase, *BHB* β-Hydroxybutyrate, *CHO* Cholesterol, *CREA* Creatinine, *GLB* Globulin, *GLU* Glucose, *HDL-C* High density lipoprotein cholesterol, *LDL-C* Low-density lipoprotein cholesterol, *NEFA* Non-esterified fatty acid, *TG* Triglyceride, *TP* Total protein, *UREA* Urea nitrogen

^a,b^Different lowercase letters indicate significant differences (*P*< 0.05)

### Plasma antioxidant and immunity

The LG group exhibited significantly higher plasma SOD concentrations compared to other groups (*P* = 0.002). The CG group showed significantly higher ROS concentrations and OSI levels than the other groups (*P* < 0.05). GTR-supplemented groups had significantly higher GST concentrations relative to the CG group (*P* = 0.009). The LG group demonstrated significantly lower GSSG concentrations than the other groups (*P* = 0.008), and its GSH/GSSG ratio was significantly higher than that of the CG group (*P* = 0.033). Immunity indexes were not significantly affected by GTR supplementation (*P* > 0.05; Table 7).

**Table 7:** Effects of GTR addition on plasma antioxidant and immunity indexes of finishing Angus steers

Items^1^	Group			SEM	*P*-value		
	CG	LG	HG		Treatment	Linear	Quadratic
SOD, U/mL	14.96^b^	17.95^a^	15.74^b^	0.373	0.002	< 0.001	< 0.001
T-AOC, mmol/mL	0.88	0.92	0.91	0.017	0.657	0.531	0.506
CAT, U/mL	5.31	5.81	5.58	0.127	0.299	0.394	0.193
MDA, nmol/mL	0.62	0.57	0.59	0.012	0.275	0.360	0.186
GSH-Px, nmol/mL	111.33	116.31	117.35	2.219	0.514	0.283	0.684
GSH, μmol/mL	0.30	0.30	0.30	0.006	0.937	0.774	0.828
GST, nmol/mL	22.99^b^	25.36^a^	24.56^a^	0.326	0.009	0.039	0.018
GSSG, nmol/mL	0.52^a^	0.45^b^	0.49^a^	0.009	0.008	0.229	0.004
GSH/GSSG	0.58^b^	0.68^a^	0.63^ab^	0.016	0.033	0.241	0.019
ROS, IU/mL	305.45^a^	268.97^b^	268.19^b^	6.484	0.026	0.018	0.179
OSI	351.26^a^	298.29^b^	302.70^b^	9.317	0.035	0.031	0.136
IL-1β, ng/L	198.68	171.03	184.98	10.875	0.601	0.618	0.383
TNF-α, ng/L	23.10	23.40	22.97	0.595	0.959	0.931	0.783
IL-18, ng/L	50.71	42.77	45.99	1.784	0.198	0.284	0.146

^1^*CAT* Catalase, *GSH* Glutathione, *GSH-Px* Glutathione peroxidase, *GST* glutathione *S*-transferase, *GSSG* Oxidized glutathione, *IL-1β* Interleukin-1β, *IL-18* Interleukin-18, *MDA* Malondialdehyde, *OSI* Oxidative stress index, *ROS* Reactive oxygen species, *SOD* Superoxidedismutase, *T-AOC* Total antioxidant capacity, *TNF-α* Tumor necrosis factor α

^a,b^Different lowercase letters indicate significant differences (*P*< 0.05)

### Plasma untargeted metabolomics

**Fig. 3:**
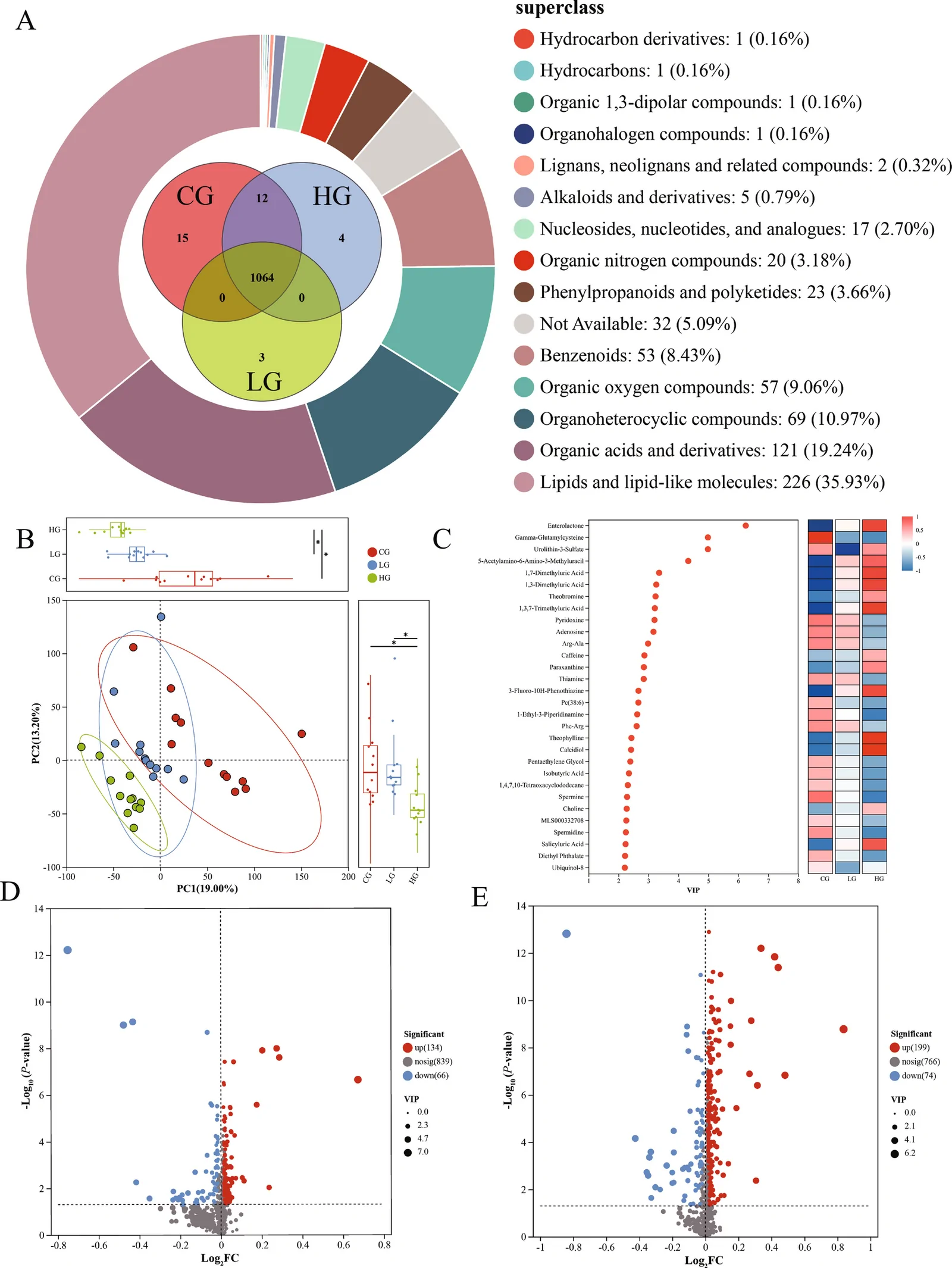
Effects of different levels of GTR on plasma metabolites in finishing Angus steers (*n* = 12 per group). **A** Metabolite HMDB compound classification (superclass level) and Venn analysis. **B** PCA of the metabolites. **C** Metabolite VIP value analysis. **D** Metabolite difference volcano plot (LG vs. CG, *P* < 0.05, VIP ≥ 1, Fold change = 1). **E** Metabolite difference volcano plot (HG vs. CG, *P* < 0.05, VIP ≥ 1, Fold change = 1)

**Fig. 4:**
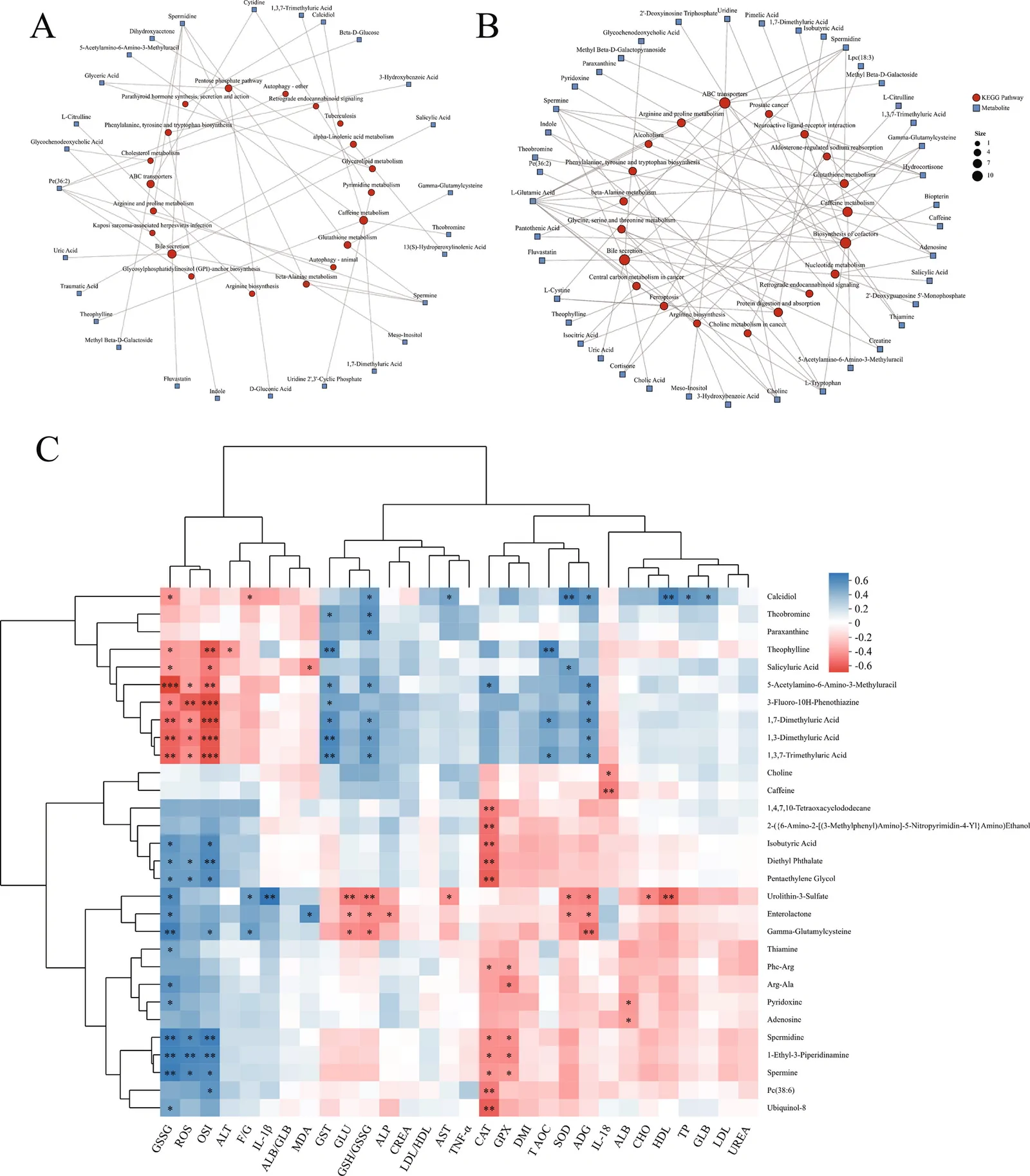
Effects of different levels of GTR on plasma metabolic pathways and correlations in finishing Angus steers. **A** KEGG enrichment analysis network diagram (LG vs. CG). **B** KEGG enrichment analysis network diagram (HG vs. CG). **C** Correlation heatmap between Top 30 metabolites and metabolic parameters. Significance levels: ^*^P < 0.05, ^**^P < 0.01, ^***^P < 0.001

A total of 1,128 metabolites were characterized in the present study, with 1,064 metabolites common to all groups. After data preprocessing, 1,039 metabolites remained (445 in positive ion mode and 594 in negative ion mode), and 640 of these were annotated against the HMDB database. At the superclass level, the three most predominant classes were lipids and lipid-like molecules (35.93%), organic acids and derivatives (19.24%), and organoheterocyclic compounds (10.97%; Fig. 3A). PCA demonstrated a distinct separation among the groups, indicating that GTR supplementation significantly influenced the plasma metabolite profile of Angus steers (Fig. 3B). Based on VIP analysis, the top three discriminatory plasma metabolites were identified as enterolactone (VIP = 6.25), gamma-glutamylcysteine (VIP = 4.99), and urolithin-3-sulfate (VIP = 4.99). Their relative abundances across the experimental groups are shown in Fig. 3C. Subsequent screening identified 200 differential metabolites in the LG group compared with the CG group, of which 134 were upregulated and 66 were downregulated (Fig. 3D). In the HG group, 273 differential metabolites were identified relative to the CG group, including 199 upregulated and 74 downregulated metabolites (Fig. 3E). KEGG enrichment analysis was subsequently performed to identify functional pathways associated with the differential metabolites. Compared with the CG group, the LG group showed enrichment in pathways such as Caffeine Metabolism, Pentose Phosphate Pathway, and GSH Metabolism (Fig. 4A). The HG group was enriched in pathways including Biosynthesis of Cofactors, Caffeine Metabolism, and GSH Metabolism (Fig. 4B). Correlation heatmap analysis revealed that enterolactone was significantly negatively correlated with GSSG and MDA, and significantly positively correlated with GLU, GSH/GSSG ratio, ALP, SOD, and ADG (*P* < 0.05). Gamma-glutamylcysteine showed significant negative correlations with GSSG, AST, and F/G, and significant positive correlations with GLU, GSH/GSSG ratio, and ADG. Pentaethylene Glycol showed significant negative correlations with GSSG, ROS, and OSI, and a significant positive correlation with CAT (*P* < 0.05; Fig. 4C).

### Mechanistic insights through integrated omics analysis

**Fig. 5:**
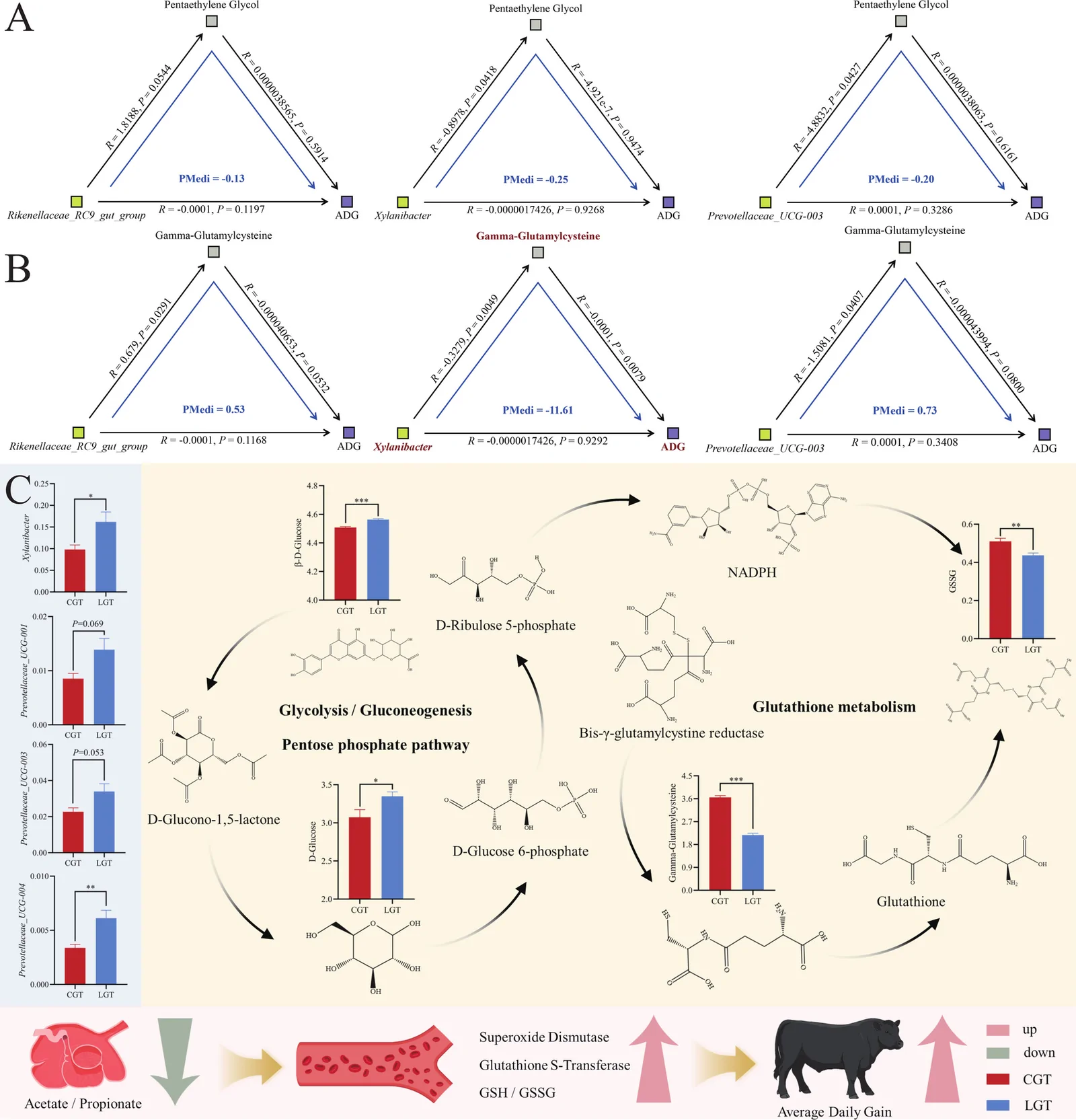
Mediation analysis and differential KEGG pathway analysis (LG vs. CG). **A** Mediation effects of top 3 differential genera on pentaethylene glycol-associated ADG changes. **B** Mediation effects of top 3 differential genera on gamma-glutamylcysteine-associated ADG changes. **C** Linking differential microbiota and metabolic pathways to growth performance. Significance levels: ^*^P < 0.05, ^**^P < 0.01, ^***^P < 0.001

This study investigated the mediating role of differential metabolites in the relationship between genus-level bacteria and growth performance. A significant mediation effect of gamma-glutamylcysteine was observed in the association between *Xylanibacter* and ADG (PMedi = −11.61) (Fig. 5A and B). Based on these findings, the mechanism of action of GTR supplementation in Angus steers was further elucidated. This mechanism involves the modulation of rumen bacterial genera and fermentation patterns, subsequent alterations in plasma metabolite profiles, and ultimate effects on metabolic function and ADG (Fig. 5C). Integrating these results with physiological indicators that in the LG group, gamma-glutamylcysteine and glutathione disulfide were significantly downregulated, whereas β-D-glucose and D-glucose were significantly upregulated. These findings demonstrated that dietary supplementation with 75 g/steer/d GTR modulated metabolic pathways including glutathione metabolism, glycolysis/gluconeogenesis, and the pentose phosphate pathway. Thereby this modulation enhances NADPH generation through energy metabolism-related pathways, thereby facilitating efficient GSSG reduction and maintaining a high GSH/GSSG ratio. Consequently, the improved nutrient utilization and enhanced antioxidant capacity contributed to the improved growth performance of Angus steers.

## Discussion

This study comprehensively assessed the impacts of dietary GTR inclusion on growth performance, ruminal fermentation characteristics, and microbial community, as well as plasma antioxidant status and metabolic profiles in finishing Angus steers. Our integrated multi-omics analysis revealed a “microbiota–metabolite–host” axis mechanism underlying the GTR-mediated regulation of growth performance. The results showed that although dietary GTR supplementation linearly increased feed costs with increasing dosage, supplementation at 75 g/steer/d tended to improve ADG and profitability in finishing beef cattle, whereas the 150 g/steer/d dosage did not yield such benefits. As a typical polyphenol-rich, locally sourced feed ingredient, GTR, when supplemented at an appropriate level, can improve growth performance sufficiently to offset the additional feed cost and ultimately enhance economic returns.

Under the present experimental conditions, dietary GTR supplementation did not significantly reduce the apparent digestibility of nutrients. Conversely, supplementation at 75 g/steer/d tended to increase the DOM, which may partly contribute to the tendency toward a lower A/P. Notably, the quadratic trend for DOM paralleled that of ADG, suggesting that improved nutrient digestibility may partially account for the growth response. To further elucidate the impact of GTR on rumen fermentation, we examined its effect on the rumen microbial composition. The results demonstrated that GTR supplementation significantly altered the rumen microbial community structure, notably enriching bacterial genera such as *Xylanibacter* and *Prevotellaceae_UCG-003*. These key propionate-producing bacteria are known to convert carbohydrates into succinate and propionate for host utilization [24]. Consistently, Cheng et al. reported that dietary supplementation with polyphenol‑rich grape pomace increased the relative abundance of propionate‑producing *Prevotella* in lambs [25]. Moreover, specific gut microbiota have been shown to regulate host energy metabolism through carbohydrate‑active enzymes [26]. Concurrently, the relative abundance of *Rikenellaceae_RC9_gut_group*, a key acetate-producing genus, was significantly reduced in the GTR‑supplemented groups [27]. This shift in the microbial community likely represents the underlying mechanism driving the changes in rumen fermentation patterns and apparent nutrient digestibility. Similarly, TPS supplementation decreased the ruminal A/P both in vivo and in vitro, an effect potentially attributable to alterations in the relative abundances of *Rikenellaceae_RC9_gut_group*, *Ruminococcaceae_NK4A214_group*, and *Butyrivibrio_2* [9]. Another study reported that 0.4% TPS reduced methane emissions and increased propionate concentration in dairy cows by decreasing total bacteria and *Ruminococcus albus* [28]. However, dietary supplementation with 150 g/steer/d GTR in this study did not significantly affect ruminal fermentation or apparent nutrient digestibility. This lack of effect may be attributed to the dose-dependent effects of polyphenols on the rumen environment. It has been reported that brown algal polyphenols can impair feed degradability by inhibiting fibrolytic bacteria [29]. A recent meta-analysis further indicated that excessive polyphenols universally suppress Gram-positive bacteria and ciliated protozoa [30]. In the present study, the lower relative abundances of *Xylanibacter* and *Prevotellaceae* in the HG group compared with the LG group are consistent with this notion. Therefore, further investigation is required to determine the optimal supplemental levels of specific polyphenol compounds in ruminants. Furthermore, some Gram‑negative bacteria can produce quorum‑sensing signaling molecules that directly impair host intestinal barrier function [31]. Whether excessive polyphenols inadvertently affect the balance of such bacterial metabolites in the rumen merits further investigation. Despite comparable alterations in rumen fermentation parameters, the associated changes in rumen microbial communities were inconsistent across studies. This inconsistency is likely due to functional redundancy of the microbiota and differences in experimental conditions. Future studies should further explore the mechanisms driving these observations.

To further investigate the impact of GTR on energy metabolism in beef cattle, we analysed plasma biochemical parameters. As an important ketone body, BHB plays a critical role in energy supply, signaling pathway regulation, and gut microbiota modulation [32]. BHB can be converted to acetyl-CoA and enter the TCA cycle, thereby decreasing the NADP/NADPH ratio and upregulating the expression of antioxidant stress‑related genes such as metallothionein [33]. Increased ruminal butyrate production has been proposed to elevate circulating BHB concentrations [34]. In the present study, ruminal butyrate and serum BHB concentrations were higher in the GTR‑supplemented groups than in the control group, a pattern compatible with this notion. We therefore interpret these metabolic changes as a coordinated response associated with both improved growth performance and the activation of the antioxidant system [32, 35]. The elevation in GLU levels was accompanied by a reduction in the ruminal A/P, indicating that GTR enhanced the supply of gluconeogenic precursors through modulation of rumen fermentation [36]. This is consistent with reports that moderate hyperglycemia and reduced insulin sensitivity in finishing beef cattle represent a metabolic adaptation to support rapid growth [37, 38]. Further supporting this interpretation, plasma lipid levels were also elevated. As a key structural component of cell membranes and a precursor for steroid hormone synthesis, CHO and its transporters, including LDL-C that delivers CHO to peripheral tissues and HDL-C that mediates reverse CHO transport, showed concurrent increases [39]. This coordinated elevation suggests that GTR may promote systemic lipid metabolism, consistent with observations in fattening sheep supplemented with *Rosa roxburghii *Tratt residue [40]. Although elevated AST and ALP activities are traditionally regarded as indicators of liver stress, the moderate increases observed in rapidly growing finishing cattle may reflect heightened metabolic activity rather than pathological damage [41, 42].

Beyond its effects on energy metabolism, GTR may also influence systemic metabolic load with plasma markers of oxidative stress and antioxidant capacity. Green tea and its polyphenols are well-characterized for their antioxidant, anti-inflammatory, immunomodulatory, and gut microbiota-modulating properties [43, 44]. The present study demonstrated that supplementation of finishing beef cattle with an optimal dose of GTR alleviated oxidative stress via activation of the GSH system. Specifically, the LG group exhibited significantly higher plasma SOD and GST activities, along with significantly lower ROS and OSI levels, and a markedly increased GSH/GSSG ratio. In the GSH antioxidant system, GSH scavenges peroxides through GSH-Px-mediated catalysis, resulting in its oxidation to GSSG. GSSG is then recycled back to GSH by glutathione reductase, which utilizes NADPH as the essential reducing cofactor [45]. This closed-loop recycling constitutes the biochemical underpinning of the system’s antioxidant function. As a critical marker of cellular redox homeostasis, an increased GSH/GSSG ratio denotes improved antioxidant capacity [46]. These results indicated that GTR selectively enhanced the antioxidant arm mediated by GST and glutathione recycling, rather than through altered GSH-Px activity. Notably, GST is primarily responsible for the detoxification of electrophilic substances, whereas GSH-Px is predominantly involved in the scavenging of peroxides [47]. Combined with the decreased levels of ROS and MDA observed in the present study, these results indicated that GTR exerted its antioxidant effects preferentially by enhancing the host’s detoxification capacity against electrophilic substances, rather than by directly activating the peroxide scavenging pathway [48]. Previous work has shown that polyphenols regulate GSH synthesis and cellular redox potential, promoting the reduction of GSSG to GSH by glutathione reductase. Polyphenols and polyphenol-rich feeds have been extensively investigated in both monogastric and ruminant livestock. In monogastrics, polyphenols commonly enhance antioxidant capacity by elevating hepatic and plasma GSH content or GSH-Px activity [49, 50]. However, findings in ruminants remain inconsistent. For instance, *Perilla frutescens* seeds supplementation modified serum immune profiles and improved antioxidant capacity in lambs, while wine grape pomace feeding increased serum CAT activity and T-AOC in finishing cattle [4, 51]. Further research is warranted to elucidate the precise pathways underlying polyphenol-mediated antioxidant defense in ruminants.

Plasma metabolomics provided further mechanistic insights into the aforementioned findings. KEGG analysis of differential metabolites revealed that gamma-glutamylcysteine, a critical intermediate in GSH biosynthesis, was significantly downregulated in the LG group, suggesting accelerated conversion to GSH [45]. Although plasma GSH concentrations remained unchanged, the marked elevation in GST activity indicated a globally activated GSH metabolism. Polyphenols can enhance the cellular antioxidant system through GST induction [52]. This effect may be attributable to Nrf2-mediated upregulation of key antioxidant enzymes, especially GST [13]. Concurrently, the increase in GLU, the primary substrate of the Pentose Phosphate Pathway, may enhance NADPH generation, which is essential for the GSH reductase-driven recycling of GSSG to GSH [53]. The simultaneous elevation of GLU and the GSH/GSSG ratio suggests a functional coupling between energy metabolism and redox homeostasis. Another key finding was that plasma Enterolactone, a microbial metabolite derived from polyphenols, was significantly elevated in the GTR‑supplemented group. Enterolactone potentiates the GSH antioxidant system through Nrf2 activation and modulates host energy metabolism by regulating gut microbiota [54, 55]. In ruminants, *Prevotella* convert the precursor secoisolariciresinol diglucoside into enterolactone [56]. Elevated ruminal and milk enterolactone levels in flaxseed‑supplemented cows have been accompanied by upregulation of *Nrf2* mRNA and reduced lipid peroxidation [57]. These findings suggest that GTR supplementation not only enriches propionate‑producing bacteria, but also promotes the production of bioactive lignan metabolites, thereby contributing to systemic antioxidant capacity. BHB concentration was positively correlated with hepatic SOD2 expression and blood SOD activity in dairy cows, which is consistent with the findings of the present study [35]. The coordinated alterations across these multiple indices highlight the systemic and integrative mechanism of action of GTR, particularly in the LG group. Notably, approximately 64% of blood metabolites are significantly correlated with the host gut microbiome [58]. Moreover, diet can shape the gut microbiota, thereby altering serum metabolite profiles and ultimately influencing host physiological indicators [59]. In livestock production, the “microbiota–metabolite–host” axis highlights the critical interplay among rumen microbiota, blood metabolites, and overall animal health and performance [60]. Mediation analysis identified gamma-glutamylcysteine as a significant mediator linking *Xylanibacter* abundance to ADG. This supports a model in which GTR supplementation in finishing Angus steers enriches propionate-producing bacteria, modulates rumen fermentation, and increases GLU concentration via gluconeogenesis. The elevated GLU fuels the Pentose Phosphate Pathway to generate NADPH, thereby promoting GSSG reduction to GSH, enhancing antioxidant capacity, and ultimately improving growth performance.

In summary, this study demonstrated that dietary GTR supplementation enriches propionate-producing bacteria, particularly *Xylanibacter*, thereby modulating ruminal fermentation patterns and increasing the supply of gluconeogenic substrates. This process subsequently elevates plasma GLU concentrations. The GLU derived from this metabolic shift can be channeled into the Pentose Phosphate pathway to generate NADPH, a critical cofactor that supports the reductive regeneration of the GSH system. Concurrently, the accelerated utilization of gamma-glutamylcysteine and the activation of GST synergistically enhance the overall antioxidant capacity. Ultimately, dietary supplementation with 75 g/steer/d GTR can improve the overall health status and growth performance of beef cattle through the mediation of the “microbiota–metabolite–host” axis. These findings provide a scientific basis for the value-added use of tea by-products in ruminant nutrition, offering a novel strategy for green and efficient beef cattle production while supporting the “One Health” goal for livestock. However, this study has several limitations. Owing to the relatively short experimental duration, the long-term effects of dietary GTR supplementation were not assessed. Post-slaughter tissue-level changes were not evaluated. In addition, the multi-omics analyses are correlation-based and therefore cannot establish causality. Future research should elucidate the molecular mechanisms by which GTR enhances antioxidant capacity and employ multi-omics approaches, such as gut microbiome and liver transcriptomics, to strengthen the mechanistic evidence.

## Conclusion

Dietary supplementation with 75 g/steer/d green tea residue improved growth performance and antioxidant capacity in finishing Angus steers. These beneficial effects were mediated through the “microbiota–metabolite–host” axis. Green tea residue reshaped the ruminal microbiota and enriched propionate-producing genera, especially *Xylanibacter*. This shift in microbial composition modulated plasma gamma-glutamylcysteine levels and activated the glutathione antioxidant system. This improved systemic redox homeostasis and ultimately enhanced growth efficiency. Furthermore, this supplementation level achieved the greatest economic profitability. The present study validates the application value of tea by-products as functional feed additives and provides a sustainable solution for improving livestock productivity.

## Supplementary Information


Additional file 1. Flavonoid composition and content of green tea residue.

## Data Availability

The original contributions made in this study are incorporated within the article. For any further inquiries, please direct them to the corresponding author.

## Abbreviations

ADF: Acid detergent fiber
ADG: Average daily gain
ALB: Albumin
ALP: Alkaline phosphatase
ALT: Alanine aminotransferase
A/P: Acetate to propionate ratio
AST: Aspartate aminotransferase
BHB: β-Hydroxybutyrate
BW: Body weight
CAT: Catalase
CG: Control group
CHO: Cholesterol
CP: Crude protein
CREA: Creatinine
DDGS: Distillers dried grains with solubles
DM: Dry matter
DMI: Dry matter intake
DOM: Organic matter apparent digestibility
EE: Ether extract
F/G: Feed-to-gain ratio
GLB: Globulin
GLU: Glucose
GSH: Glutathione
GSH-Px: Glutathione peroxidase
GSSG: Oxidized glutathione
GST: Glutathione S-transferase
GTR: Green tea residue
HDL-C: High-density lipoprotein cholesterol
HG: High-dose GTR group
HMDB: Human metabolome database
IL-18: Interleukin-18
IL-1β: Interleukin-1β
LDL-C: Low-density lipoprotein cholesterol
LG: Low-dose GTR group
MDA: Malondialdehyde
NDF: Neutral detergent fiber
NEFA: Non-esterified fatty acid
NH_3_-N: Ammonia nitrogen
OM: Organic matter
OSI: Oxidative stress index
PCA: Principal component analysis
ROS: Reactive oxygen species
SEM: Standard error of mean
SOD: Superoxide dismutase
T-AOC: Total antioxidant capacity
TG: Triglyceride
TNF-α: Tumor necrosis factor α
TP: Total protein
TPS: Tea polyphenols
TVFA: Total volatile fatty acids
UREA: Urea nitrogen
VIP: Variable importance in projection
WSC: Water soluble carbohydrates

## References

[1] Govoni C, Ottoboni M, Manoni M, Pinotti L, Rulli MC. Upcycling former food products in livestock diets: a one health approach to prevent resource-depleting farming systems. Resour Conserv Recy. 2025;223:108536. 10.1016/j.resconrec.2025.108536.

[2] Wang J, Deng L, Chen M, Che Y, Li L, Zhu L, et al. Phytogenic feed additives as natural antibiotic alternatives in animal health and production: a review of the literature of the last decade. Anim Nutr. 2024;17:244–64. 10.1016/j.aninu.2024.01.012. 38800730 PMC11127233

[3] You W, Cheng H, Hu X, Song E, Jiang F. Capsaicin modulates ruminal fermentation and bacterial communities in beef cattle with high-grain diet-induced subacute ruminal acidosis. Microorganisms. 2025;13(1):84. 10.3390/microorganisms13010084. 39858852 PMC11767826

[4] Yu Y, Zhang B, Jiang X, Cui Y, Shang Y, Jin Y, et al. Perilla frutescens seeds enhance lamb immunity and antioxidant capacity via the microbiota-gut-liver-muscle axis. J Anim Sci Biotechnol. 2026;17:1. 10.1186/s40104-025-01317-3. 41485094 PMC12765310

[5] Dai D, Dong C, Kong F, Wang S, Wang S, Wang W, et al. Dietary supplementation of *Scutellariae radix* flavonoid extract improves lactation performance in dairy cows by regulating gastrointestinal microbes, antioxidant capacity and immune function. Anim Nutr. 2025;20:499–508. 10.1016/j.aninu.2024.11.019. 40092352 PMC11909456

[6] Yan Z, Zhong Y, Duan Y, Chen Q, Li F. Antioxidant mechanism of tea polyphenols and its impact on health benefits. Anim Nutr. 2020;6(2):115–23. 10.1016/j.aninu.2020.01.001. 32542190 PMC7283370

[7] Wu X, Wang Y, Wang D, Wang Z, Yang M, Yang L, et al. Formation of EGCG oxidation self-assembled nanoparticles and their antioxidant activity in vitro and hepatic REDOX regulation activity in vivo. Food Funct. 2024;15(4):2181–96. 10.1039/d3fo05309a. 38315103

[8] Hao L, Zhao Z, Zhou H, Wen L, Liu X, Yu Y, et al. Resource utilization of tea waste in biochar and other areas: current status, challenges and future prospects. J Environ Manage. 2025;377:124569. 10.1016/j.jenvman.2025.124569. 39983568

[9] Teng Z, Liu S, Zhang L, Zhang L, Liu S, Fu T, et al. Tea polyphenols inhibit methanogenesis and improve rumen epithelial transport in dairy cows. Animals. 2024;14(17):2569. 10.3390/ani14172569. 39272354 PMC11394105

[10] Gheshlagh NS, Paya H, Taghizadeh A, Mohammadzadeh H, Palangi V, Mehmannavaz Y. Comparative effects of extracted polyphenols from black and green tea wastes on in-vitro fermentability of feed ingredients. Semin-Cienc Agrar. 2021;42(3Supl1):2005–22. 10.5433/1679-0359.2021v42n3Supl1p2005.

[11] Qiu Q, Wei X, Zhang L, Li Y, Qu M, Ouyang K. Effect of dietary inclusion of tea residue and tea leaves on ruminal fermentation characteristics and methane production. Anim Biotechnol. 2023;34(4):825–34. 10.1080/10495398.2021.1998092. 34730482

[12] Luo W, Tan Q, Li H, Ye T, Xiao T, Tian X, et al. Effects of different levels of green tea powder on performance, antioxidant activity, egg mass, quality, and cecal microflora of chickens. Animals. 2024;14(20):3020. 10.3390/ani14203020. 39457950 PMC11505839

[13] Khan IM, Gul H, Khan S, Nassar N, Khalid A, Swelum AA, et al. Green tea polyphenol epigallocatechin-3-gallate mediates an antioxidant response via Nrf2 pathway in heat-stressed poultry: A review. Poultry Sci. 2025;104(5):105071. 10.1016/j.psj.2025.105071. 40157268 PMC11995091

[14] Bešlo D, Došlić G, Agić D, Rastija V, Šperanda M, Gantner V, et al. Polyphenols in ruminant nutrition and their effects on reproduction. Antioxidants (Basel). 2022;11(5):970. 10.3390/antiox11050970. 35624834 PMC9137580

[15] National Academies of Sciences, Engineering, and Medicine. Nutrient requirements of beef cattle: eighth revised edition. Washington, DC: The National Academies Press; 2016. 38386771

[16] National Technical Committee on Tea of Standardization Administration of China. Determination of total polyphenols and catechins content in tea: GB/T 8313–2018. Beijing: Standards Press of China; 2018.

[17] Mcdonald P, Henderson AR. Determination of water-soluble carbohydrates in grass. J Sci Food Agr. 1964;15(6):395–8. 10.1002/jsfa.2740150609.

[18] Van Soest PJ, Robertson JB, Lewis BA. Methods for dietary fiber, neutral detergent fiber, and nonstarch polysaccharides in relation to animal nutrition. J Dairy Sci. 1991;74(10):3583–97. 10.3168/jds.S0022-0302(91)78551-2. 1660498

[19] AOAC International. Official methods of analysis. 18th ed. Gaithersburg, MD, USA: AOAC International; 2006.

[20] Lee SB, Lee KW, Wang T, Lee JS, Lee HG. Administration of encapsulated L-tryptophan improves duodenal starch digestion and increases gastrointestinal hormones secretions in beef cattle. Asian-Australas J Anim Sci. 2020;33(1):91–9. 10.5713/ajas.19.0498. PMC6946987 31902185

[21] Keulen JV, Young BA. Evaluation of acid-insoluble ash as a natural marker in ruminant digestibility studies. J Anim Sci. 1977;44(2):282–7. 10.2527/jas1977.442282x.

[22] Weatherburn MW. Phenol-hypochlorite reaction for determination of ammonia. Anal Chem. 1967;39(8):971–4. 10.1021/ac60252a045.

[23] He Y, Wang H, Yu Z, Niu W, Qiu Q, Su H, et al. Effects of the gender differences in cattle rumen fermentation on anaerobic fermentation of wheat straw. J Clean Prod. 2018;205:845–53. 10.1016/j.jclepro.2018.09.156.

[24] Murovec U, Accetto T. Transcriptomic analysis of polysaccharide utilization loci reveals substrate preferences in ruminal generalists *Segatella bryantii* TF1-3 and *Xylanibacter ruminicola* KHP1. BMC Genomics. 2024;25(1):495. 10.1186/s12864-024-10421-z. 38769483 PMC11107044

[25] Cheng X, Du X, Liang Y, Degen AA, Wu X, Ji K, et al. Effect of grape pomace supplement on growth performance, gastrointestinal microbiota, and methane production in Tan lambs. Front Microbiol. 2023;14:1264840. 10.3389/fmicb.2023.1264840. 37840727 PMC10569316

[26] Ma L, Tao S, Song T, Lyu W, Li Y, Wang W, et al. *Clostridium butyricum* and carbohydrate active enzymes contribute to the reduced fat deposition in pigs. Imeta. 2024;3(1):e160. 10.1002/imt2.160. PMC10989082 38868506

[27] Miao H, Yin Z, Yang K, Gu P, Ren X, Zhang Z. Bioaugmentation with rumen fluid to improve acetic acid production from kitchen waste. Water Air Soil Pollut. 2024;235(11):683. 10.1007/s11270-024-07484-9.

[28] Zhao R, Sun J, Lin Y, Yan H, Zhang S, Huo W, et al. Effects of dietary tannic acid and tea polyphenol supplementation on rumen fermentation, methane emissions, milk protein synthesis and microbiota in cows. Microorganisms. 2025;13(8):1848. 10.3390/microorganisms13081848. 40871352 PMC12388258

[29] Pandey D, Hansen HH, Dhakal R, Aryal N, Rai SP, Sapkota R, et al. Interspecies and seasonal variations in macroalgae from the Nordic region: Chemical composition and impacts on rumen fermentation and microbiome assembly. J Clean Prod. 2022;363:132456. 10.1016/j.jclepro.2022.132456.

[30] Vasta V, Daghio M, Cappucci A, Buccioni A, Serra A, Viti C, et al. Invited review: plant polyphenols and rumen microbiota responsible for fatty acid biohydrogenation, fiber digestion, and methane emission: experimental evidence and methodological approaches. J Dairy Sci. 2019;102(5):3781–804. 10.3168/jds.2018-14985. 30904293

[31] Xiao Y, Zou H, Li J, Song T, Lv W, Wang W, et al. Impact of quorum sensing signaling molecules in Gram-negative bacteria on host cells: current understanding and future perspectives. Gut Microbes. 2022;14(1):2039048. 10.1080/19490976.2022.2039048. PMC8865250 35188058

[32] Zhuang Y, Liu G, Jiang C, Abdelsattar MM, Fu Y, Li Y, et al. Dietary β-hydroxybutyrate sodium alters rumen microbiome and nutrient metabolism in the rumen epithelium of young goats. J Integr Agric. 2026;25(4):1619–35. 10.1016/j.jia.2024.11.016.

[33] Sharma R, Ramanathan A. The aging metabolome-biomarkers to hub metabolites. Proteomics. 2020;20(5–6):e1800407. 10.1002/pmic.201800407. 32068959 PMC7117067

[34] Herrick KJ, Hippen AR, Kalscheur KF, Schingoethe DJ, Ranathunga SD, Anderson JL, et al. Infusion of butyrate affects plasma glucose, butyrate, and β-hydroxybutyrate but not plasma insulin in lactating dairy cows. J Dairy Sci. 2018;101(4):3524–36. 10.3168/jds.2017-13842. 29409601

[35] Grzybowska D, Żarczyńska K, Sobiech P, Brym P, Tobolski D. Persistently high concentrations of β-hydroxybutyrate affect hepatic SOD2 expression and blood SOD activity in high-yielding dairy cows. BMC Vet Res. 2025;21:12. 10.1186/s12917-024-04464-3. PMC11716416 39789575

[36] Pang R, Xiao X, Mao T, Yu J, Huang L, Xu W, et al. The molecular mechanism of propionate-regulating gluconeogenesis in bovine hepatocytes. Anim Biosci. 2023;36(11):1693–9. 10.5713/ab.23.0061. 37402451 PMC10623044

[37] Foote AP, Salisbury CM, King ME, Rathert-Williams AR, McConnell HL, Beck MR. Association of glucose metabolism and insulin resistance with feed efficiency and production traits of finishing beef steers. J Anim Sci. 2024;102:skae050. 10.1093/jas/skae050. PMC10926941 38401157

[38] Foote AP, Salisbury C, Beck MR, Montgomery D, Rathert-Williams AR, King ME. PSII-B-12 association of glucose metabolism with feed intake, growth, and efficiency of beef steers. J Anim Sci. 2022;100(Suppl 3):365. 10.1093/jas/skac247.666.

[39] Cui D, Yu X, Guan Q, Shen Y, Liao J, Liu Y, et al. Cholesterol metabolism: molecular mechanisms, biological functions, diseases, and therapeutic targets. Mol Biomed. 2025;6(1):72. 10.1186/s43556-025-00321-3. 41062796 PMC12508344

[40] Li H, Song X, Wu W, Zhou C. *Rosa roxburghii* tratt residue as an alternative feed for improving growth, blood metabolites, rumen fermentation, and slaughter performance in Hu sheep. Front Vet Sci. 2024;11:1397051. 10.3389/fvets.2024.1397051. 38962702 PMC11220278

[41] Yao Y, Li Z, Qin B, Ju X, Wang L. Evaluation of the intracellular lipid-lowering effect of polyphenols extract from highland barley in HepG2 cells. Food Sci Hum Well. 2024;13(1):454–61. 10.26599/FSHW.2022.9250039.

[42] Shi C, Li Y, Yang S, Luo Y, Min S, Wang K, et al. Supplementing late-fattening Angus steers diets with yeast culture remodels gastrointestinal microbiota and promotes physiological adaptation to heat stress. Results Eng. 2026;29:109307. 10.1016/j.rineng.2026.109307.

[43] Truong VL, Jeong WS. Antioxidant and anti-inflammatory roles of tea polyphenols in inflammatory bowel diseases. Food Sci Hum Well. 2022;11(3):502–11. 10.1016/j.fshw.2021.12.008.

[44] Luo Q, Luo L, Zhao J, Wang Y, Luo H. Biological potential and mechanisms of tea’s bioactive compounds: an updated review. J Adv Res. 2024;65:345–63. 10.1016/j.jare.2023.12.004. 38056775 PMC11519742

[45] Tan M, Yin Y, Ma X, Zhang J, Pan W, Tan M, et al. Glutathione system enhancement for cardiac protection: pharmacological options against oxidative stress and ferroptosis. Cell Death Dis. 2023;14(2):131. 10.1038/s41419-023-05645-y. 36792890 PMC9932120

[46] Giustarini D, Colombo G, Garavaglia ML, Astori E, Portinaro NM, Reggiani F, et al. Assessment of glutathione/glutathione disulphide ratio and S-glutathionylated proteins in human blood, solid tissues, and cultured cells. Free Radic Biol Med. 2017;112:360–75. 10.1016/j.freeradbiomed.2017.08.008. 28807817

[47] Aloke C, Onisuru OO, Achilonu I. Glutathione S-transferase: A versatile and dynamic enzyme. Biochem Bioph Res Co. 2024;734:150774. 10.1016/j.bbrc.2024.150774. 39366175

[48] Averill-Bates DA. The antioxidant glutathione. Vitam Horm. 2023;121:109–41. 10.1016/bs.vh.2022.09.002. 36707132

[49] Xiao Y, Gao X, Yuan J. Substituting ethoxyquin with tea polyphenols and propyl gallate enhanced feed oxidative stability, broiler hepatic antioxidant capacity and gut health. Poult Sci. 2024;103(12):104368. 10.1016/j.psj.2024.104368. 39405832 PMC11525215

[50] Zheng Y, Li Y, Yu B, Luo Y, Huang Z, Zheng P, et al. Dietary supplementation of grape seed proanthocyanidins improves growth performance, carcass traits, and meat quality in growing-finishing pigs. Anim Nutr. 2025;20:200–10. 10.1016/j.aninu.2024.10.006. 39967699 PMC11833782

[51] Li Y, Shi C, Deng J, Qiu X, Zhang S, Wang H, et al. Effects of grape pomace on growth performance, nitrogen metabolism, antioxidants, and microbial diversity in Angus bulls. Antioxidants (Basel). 2024;13(4):412. 10.3390/antiox13040412. 38671860 PMC11047470

[52] Fiander H, Schneider H. Dietary ortho phenols that induce glutathione S-transferase and increase the resistance of cells to hydrogen peroxide are potential cancer chemopreventives that act by two mechanisms: the alleviation of oxidative stress and the detoxification of mutagenic xenobiotics. Cancer Lett. 2000;156(2):117–24. 10.1016/S0304-3835(00)00368-2. 10880760

[53] Banki K, Perl A. Cell type-specific regulation of the pentose phosphate pathway during development and metabolic stress-driven autoimmune diseases: relevance for inflammatory liver, renal, endocrine, cardiovascular and neurobehavioral comorbidities, carcinogenesis, and aging. Autoimmun Rev. 2025;24(5):103781. 10.1016/j.autrev.2025.103781. 40010622 PMC13504198

[54] Ku M, Taibi A, Wu D, Chen YT, Lopez-Dominguez L, Yang Y, et al. Altered mouse cecal microbiome-serum enterolignans relationships in response to dietary lignans ingested through whole flaxseed or flaxseed hull. Food Funct. 2026;17(1):204–15. 10.1039/d5fo03558a. 41263597

[55] Kivelä AM, Kansanen E, Jyrkkänen HK, Nurmi T, Ylä-Herttuala S, Levonen AL. Enterolactone induces heme oxygenase-1 expression through nuclear factor-e2-related factor 2 activation in endothelial cells. J Nutr. 2008;138(7):1263–8. 10.1093/jn/138.7.1263. 18567745

[56] Ghedini CP, Silva LHP, Moura DC, Brito AF. Supplementing flaxseed meal with sucrose, flaxseed oil, or both: effects on milk enterolactone, ruminal microbiota profile, production performance, and nutrient utilization in dairy cows. J Dairy Sci. 2024;107(9):6834–51. 10.3168/jds.2024-24649. 38762110

[57] Schogor ALB, Palin MF, Dos Santos GT, Benchaar C, Lacasse P, Petit HV. Mammary gene expression and activity of antioxidant enzymes and oxidative indicators in the blood, milk, mammary tissue and ruminal fluid of dairy cows fed flax meal. Br J Nutr. 2013;110(10):1743–50. 10.1017/S0007114513001220. 23578516

[58] Diener C, Dai CL, Wilmanski T, Baloni P, Smith B, Rappaport N, et al. Genome-microbiome interplay provides insight into the determinants of the human blood metabolome. Nat Metab. 2022;4(11):1560–72. 10.1038/s42255-022-00670-1. 36357685 PMC9691620

[59] Zhang J, Qi H, Li M, Wang Z, Jia X, Sun T, et al. Diet mediate the impact of host habitat on gut microbiome and influence clinical indexes by modulating gut microbes and serum metabolites. Adv Sci. 2024;11(19):2310068. 10.1002/advs.202310068. PMC11109649 38477427

[60] Zhang C, Wang M, Liu H, Jiang X, Chen X, Liu T, et al. Multi-omics reveals that the host-microbiome metabolism crosstalk of differential rumen bacterial enterotypes can regulate the milk protein synthesis of dairy cows. J Animal Sci Biotechnol. 2023;14:63. 10.1186/s40104-023-00862-z. PMC10169493 37158919

